# Bioactive compounds from an endophytic fungi *Nigrospora aurantiaca*

**DOI:** 10.1186/s40529-021-00324-7

**Published:** 2021-10-26

**Authors:** Safwan Safwan, George Hsiao, Tzong-Huei Lee, Ching-Kuo Lee

**Affiliations:** 1 Clinical Drug Development of Herbal Medicine, College of Pharmacy, Taipei Medical University, Taipei, 11031 Taiwan; 2 Department of Pharmacy, Faculty of Health Science, University of Muhammadiyah Mataram, Mataram, 83127 Indonesia; 3 Graduate Institute of Medical Sciences, College of Medicine, Taipei Medical University, Taipei, 11031 Taiwan; 4 Department of Pharmacology, School of Medicine, Taipei Medical University, Taipei, 11031 Taiwan; 5 Institute of Fisheries Science, National Taiwan University, Taipei, 10617 Taiwan; 6 School of Pharmacy, Taipei Medical University, Taipei, 11031 Taiwan

**Keywords:** *Nigrospora aurantiaca*, *Melaleuca leucadendra*, Endophytes fungi, Nitric oxide production

## Abstract

**Background:**

Many groups of fungi live as an endophyte in plants. Both published and undiscovered bioactive compounds can be found in endophytic fungi. Various biological activities of bioactive compounds from endophytic fungi had been reported, including anti-inflammatory and anticancerous effects.

The chemical investigation of biologically active compounds from endophytic fungi *Melaleuca leucadendra* Linn. have not yet been stated.

**Results:**

One new compound, namely nigaurdiol (**1**), along with five known compounds, xyloketal K (**2**), bostrycin (**3**), deoxybostrycin (**4**), xylanthraquinone (**5**), and ergosterol (**6**), were isolated from the *Melaleuca leucadendra* Linn. associated fungal strain *Nigrospora aurantiaca* ^#^TMU062. Their chemical structures were elucidated by spectroscopic data and compared with literature. All isolated compounds were evaluated for inhibitory effect of NO production in LPS-activated microglial BV-2 cells.

**Conclusions:**

Compound **6** exhibited considerable inhibitory effect on NO production with IC_50_ values of 7.2 ± 1.4 µM and the survival rate of the cells was 90.8 ± 6.7% at the concentration of 10 µM.

## Background

Endophytes are defined as microorganisms that spend at least parts of their life cycle inhabiting in its host plants without causing apparent harm to the host (Hardoim et al. 2015). Endophytic fungi is one of the potential resources for obtaining bioactive compounds because of its complex interaction with their host plants or other microorganisms within the host plants. Previous studies showed that many bioactive compounds produced by endophytic fungi exhibit antioxidant, anticancer, anti-inflammatory, antimicrobial, and other biological activities (Kumari et al. 2018; Ukwatta et al. 2020). Some of the medicinal plants have been found to rear a number of highly diversified endophytic fungi, which could even produce the same compounds as their host plants. For instance, ginkgolide B can be produced by both *Fusarium oxysporum* and its host plant *Ginkgo biloba* (Cui et al. 2012). Thus, many of the folk medicinal plants were chosen to screen the associated fungal strains with significant biological activities in the recent past.

*Melaleuca leucadendra* Linn. of the Myrtaceae family is distributed across Australia and Southeast Asia countries like Indonesia (Pujiarti et al. 2011). The leaves of this family are known to contain a high concentration of terpenes with varied quality and quantity (Keszei et al. 2008). As a folk medicine, *M. leucadendra* Linn. was reported to exhibit antioxidant, antiproliferative, and anticancer activities (Rini et al. 2012; Monzote et al. 2020). However, related researches of the endophytic fungi from *M. leucadendra* Linn. have not yet been reported. This study focuses on the bioactivity and chemical investigation of *Nigrospora aurantiaca* isolated from *M. leucadendra* Linn.

## Results and discussion

Through chemical investigation of the liquid and solid fermented products, one new compound together with five known compounds on *N. aurantiaca* (an endophytic fungi from *M. leucadendra*) were identified. By comparing with literature data, the well-known compounds were recognized as xyloketal K (**2**) (Sun et al. 2016), bostrycin (**3**) (Stevens et al. 1979; Chen et al. 2012), deoxybostrycin (**4**) (Chen et al. 2012; Wang et al. 2013), xylanthraquinone (**5**) (Sommart et al. 2008), and ergosterol (**6**) (Kawai et al. 2018).

**Fig. 1:**
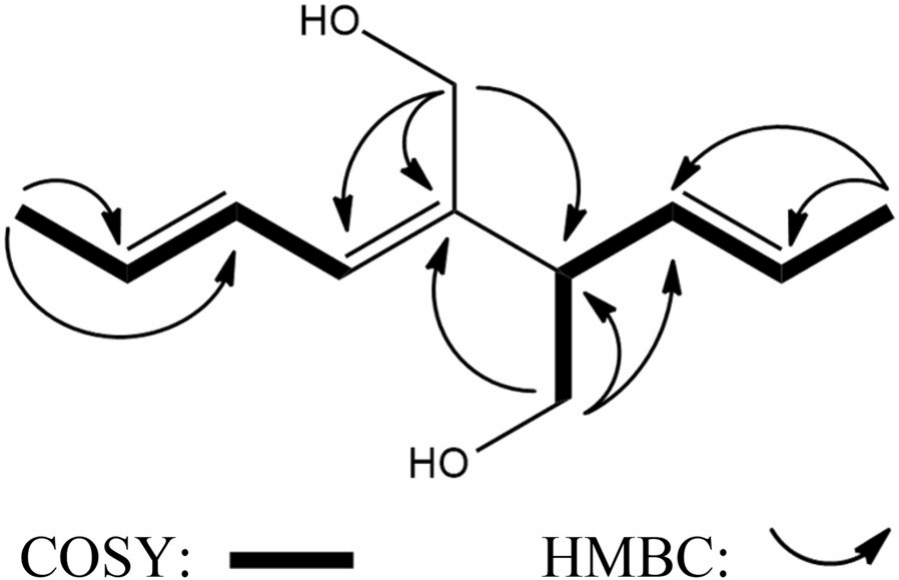
Key HMBC and COSY correlations for compound **1**

**Fig. 2:**
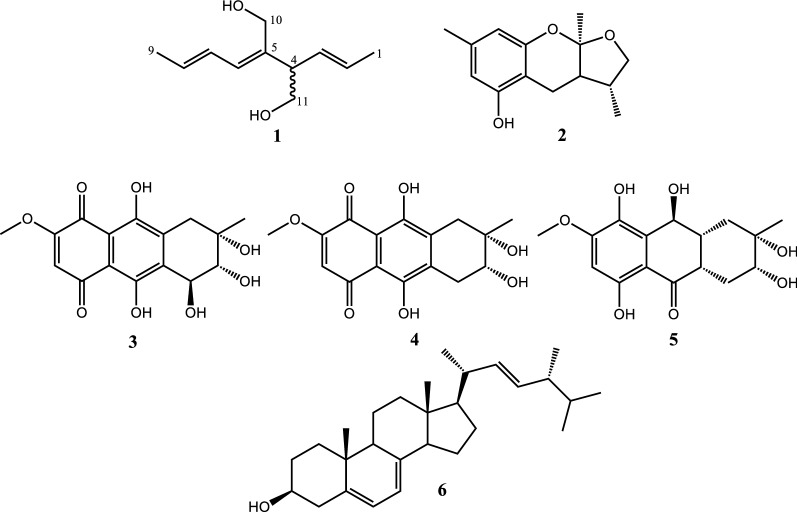
Chemical structures of compounds **1**–**6**

Compound **1**, a colorless oil, was determined to have a molecular formula of C_11_H_18_O_2_, ([M + H]^+^ *m*/*z* 183.1381, calcd 183.1380) by HRESIMS analysis and evidenced by ^13^C NMR spectrum. The IR spectrum confirmed the presence of hydroxy and olefinic functionalities at 3334 and 1646 cm^–1^, respectively. The ^1^H NMR data (CD_3_OD, 600 MHz) spectrum showed two methyl groups of δ_H_ 1.67 (3H, d, *J* = 6.2 Hz, H_3_-1) and δ_H_ 1.77 (3H, dd, *J* = 6.7, 1.2 Hz, H_3_-9); six methine signals at δ_H_ 3.00 (1H, dt, *J* = 7.0, 7.0 Hz, H-4), δ_H_ 5.43 (1H, dd, *J* = 16.4, 7.0 Hz, H-3), δ_H_ 5.52 (1H, dq, *J* = 16.4, 6.2 Hz, H-2), δ_H_ 5.70 (1H, dq, *J* = 14.8, 6.7 Hz, H-8), δ_H_ 5.94 (1H, d, *J* = 11.0 Hz, H-6), and δ_H_ 6.43 (1H, ddq, *J* = 14.8, 11.0, 1.2 Hz, H-7); and two oxygenated methylene signals at δ_H_ 3.55 and 3.63 (each 1H, dd, *J* = 10.7, 7.0 Hz, H_2_-11) and δ_H_ 4.14 and 4.18 (each 1H, d, *J* = 12.0 Hz, H_2_-10). The DEPT ^13^C NMR in combination with the ^13^C NMR (CD_3_OD) and HSQC spectrum of **1** contained 11 carbon signals corresponding to two methyls at δ_C_ 16.8 (C-1) and 17.00 (C-9); six methines at δ_C_ 50.7 (C-4), 126.2 (C-2), 126.9 (C-7), 129.2 (C-6), 129.6 (C-8), and 130.7 (C-3); and two methylenes at δ_C_ 58.1 (C-10) and 64.4 (C-11). The COSY spectrum (Fig. 1) revealed contiguous protons of H-9/H-8/H-7/H-6 and H-1/H-2/H-3 /H-4 /H-11. Key cross-peaks of HMBC spectrum (Fig. 1) including δ_H_ 4.18 (H_2_-10)/δ_C_ 137.6 (C-5), 50.4 (C-4), and 129.2 (C-6); δ_H_ 3.63 (H_2_-11)/δ_C_ 137.6 (C-5), 50.4 (C-4), and 130.7 (C-3); δ_H_ 3.00 (H-4)/δ_C_ 130.7 (C-3), 137.6 (C-5), 129.2 (C-6), and 126.2 (C-2) were observed. The structure of **1** was thus determined as shown in Fig. 2, and named nigaurdiol. The chemical skeleton of **1** has not been reported previously; it could be a recemate since the optical rorational value of **1** was close to zero.

All six isolates were evaluated for their inhibitory effects on nitric oxide (NO) production and cytotoxicity in LPS-activated microglial BV-2 cells. For positive control, curcumin was used with an IC_50_ value of 6.0 ± 0.3 µM. Compounds **3**, **4**, and **6** showed potently inhibitory effects with IC_50_ value of 2.3 ± 0.3, 2.5 ± 0.5, and 7.2 ± 1.4, respectively; however, compounds **3** and **4** exhibited significant cytotoxicity against microglial BV-2 cell with viabillities of 10.7 ± 0.8 and 11.3 ± 1.3% (10 µM), respectively. Furthermore, compound **6** showed no significant cytotoxic effect with the survival of cells at concentration 10 µM of 90.8 ± 6.7%. Compounds **1**, **2** and **5** showed weak inhibitory effects and no cytotoxic activity (Table 1). Ergosterol (**6**) is the major sterol endogenously produced by fungi and protozoa with diverse bioactivities—including anti-inflammatory, anti-cancer, and immune-modulatory effects (Lee et al. 2017; Papoutsis et al. 2020).

**Table 1:** IC_50_ and cell viabillity values of compounds in BV-2 microgial cells

Compounds	IC_50_ (µM)	Cell viabillity (%)
1	32.2 ± 3.3	102.6 ± 8.8
2	30.1 ± 3.0^*^	98.3 ± 7.6
3	2.3 ± 0.3^***^	10.7 ± 0.8^***^
4	2.5 ± 0.5^***^	11.3 ± 1.3^***^
5	32.1 ± 6.7	102.8 ± 6.9
6	7.2 ± 1.4^***^	90.8 ± 6.7
R	1.4 ± 0.8	100.0 ± 0
V	38.2 ± 4.7^###^	–
Curcumin	6.0 ± 0.3	–

Data are as the mean ± SD (*n* = 3)

^*^p < 0.05, ^**^p < 0.01, and ^***^p < 0.001 compared with the stimulation (V); ^###^p < 0.001 compared with the resting (R)

## Conclusions

In this report, we have identified one new compound, nigaurdiol (**1**), along with five known compounds **2** – **6** from an endophytic fungus (identified as *Nigrospora aurantiaca* ^#^TMU062) associated with *Melaleuca leucadendra* Linn. Of the compounds identified, the chemical skeleton of nigaurdiol (**1**) is being shown for the first time. All compounds were evaluated by *in-vitr*o NO inhibitory assay in the LPS-stimulated murine BV-2 microglial cell line. The results showed potential inhibitory activities in bostrycin (**3**), deoxybostrycin (**4**), and ergosterol (**6**) than nigaurdiol (**1**), xyloketal K (**2**), and xylanthraquinone (**5**) weak inhibitory activities. Bostrycin (**3**) and deoxybostrycin (**4**) exhibited significant cytotoxicity against microglial BV-2 cell.

## Methods

### General experimental procedures

^1^H, ^13^C, DEPT, and 2D NMR were acquired on Agilent DD2 600 MHz pectrometer (Agilent Technologies, Santa Clara, CA, USA). Optical rotation was measured with a JASCO P-2000 polarimeter (Tokyo, Japan). IR spectra were recorded on a JASCO FT/IR 4100 spectrometer (Tokyo, Japan). Sephadex LH-20 (GE Healthcare, Uppsala, Sweden) was used for open column chromatography. High-resolution mass spectrometry data was acquired using Q Exactive Plus Hybrid Quadrupole-Orbitrap Mass Spectrometer (Thermo Fisher Scientific, Bremen, Germany) coupled with the Dionex UltiMate™ 3000 RSLCnano UHPLC system (Thermo Fisher Scientific, San Jose, CA, USA). Semi-preparative HPLC experiments for compound purification were performed using HPLC pump L-7100 (Hitachi, Japan) with refractiveindex (Bischoff, Leonberg, Germany) for detector.

### Fungal material

The fungal strain *Nigrospora aurantiaca* was isolated from a healthy leaf of *Melaleuca leucadendra* linn collected in the yard of National Taiwan University and was identified by sequencing the internal transcribed spacer regions of the rDNA (ITS). A BLAST search of the sequence led to the best match of *Nigrospora aurantiaca*. Mycelium *Nigrospora aurantiaca* ^#^TMU062 was inoculated into two different media—liquid medium and solid medium. Inoculation in liquid medium was done in 5 L serum bottles, each containing 50 g of malt extract (Becton, Dickinson and Company, Sparks, USA) and 3.5 L of deionized water. The fermentation was conducted with aeration at 25–30 °C for 14 days. As for solid medium, 250 mL flasks were used—each containing 20 g of barley and 0.2 g of potato dextrose agar (Becton, Dickinson and Company, Sparks, USA). After adding 15 mL of deionized water, they were fermented for 30 days at 27–30 °C.

### Extraction and isolation

The fermented broth (9.5 L) was filtered and partitioned five times with equal volumes of EtOAc and subsequently concentrated in vacuum to obtain crude extract (5.8 g). The crude extract was re-dissolved in 50 mL MeOH to obtain MeOH layer and sediment (2.3 g). Then, the sediment was dissolved in 10 mL DMSO and purified by HPLC semipreparative reversed-phase column (Phenomenex Luna PFP, 5 μm, 10 × 250 mm, Torrance, CA, USA) eluted by 65% MeOH, 2 mL/min, to obtain **3** (*t*_R_: 12 min; 50.0 mg) and three fractions (Fr.S2-Fr.S4). Further purification of Fr.S3 on HPLC on a semipreparative reversed-phase column (Thermo Hypersil HS C18, 5 μm, 10 × 250 mm, Bellefonte, PA, USA) eluted by 50% MeOH, 2 mL/min to obtain **4** (*t*_R_: 9 min; 4.9 mg). The MeOH layer was concentrated under vacuum into 15 mL, then applied into a Sephadex LH-20 column (2.5 i.d. × 68.5 cm) eluted by MeOH with a flow rate of 2.5 mL/min to give forty-five fractions (25 mL) before combined into seven fractions as Fr.A – Fr.G based on similar compositions of TLC analysis. The Fr.B (1.3 g) and Fr.C (1.05 g) were purified by HPLC on a semipreparative reversed-phase column (Phenomenex Luna PFP, 5 μm, 10 i.d. × 250 mm, Torrance, CA, USA) eluted by MeOH (respectively, 60% and 65%) to obtain four subractions (Fr.B1-Fr.B4) and eight subfractions (Fr.C1-Fr.C8) from Fr.B dan Fr.C, respectively. Further purification of Fr.B1 and Fr.C7 by HPLC semipreparative reversed-phase column (Thermo Hypersil HS C18, 5 μm, 10 i.d. × 250 mm, Bellefonte, PA, USA) eluted by MeOH_aq_ (respectively, 30% and 50%) to give **1** (*t*_R_: 21 min; 3.2 mg), **2** (*t*_R_: 25 min; 3.5 mg) and **5** (*t*_R_: 13 min, 7.0 mg). The solid fermented products were grinded into a powder after cryodesiccation and than extracted four times with MeOH (equal volumes). The crude extracts were suspended with H_2_O and partitioned three times with EA, *n*-hexane, and *n*-butanol, respectively (equal volumes). The dried *n*-hexane extract (3.4 g) was subjected to gravity column chromatography (5 i.d. × 17 cm) with silica, eluted with *n*-*hexane*, EA, and MeOH by gradient system to yield 52 fractions, beofre combined into 12 fractions (fr.A-Fr.L) based on similar compositions of TLC analysis. Compound **6** (10.0 mg) was obtained from the recrystallization of fraction Fr.B at − 4 °C for 12 h.

### Nigaurdiol (1)

colorless oil; [α]^25^_D_ = − 1.2 (*c* 0.3, MeOH); IR (ν_max_, KBr): at 3334 and 1646 cm^−1^; HR-ESI–MS: [M + H]^+^ *m*/*z* 183.1381 (calcd. 183.1380 for C_11_H_19_O_2_); ^1^H and ^13^C NMR see Table 2.

**Table 2:** NMR data of compound **1** in CD_3_OD

Position	δ_C_	δ_H_ (*J* in Hz)
1	16.8	1.67 (d, 3H, *J* = 6.2 Hz)
2	126.2	5.52 (dq, 1H, *J* = 16.4, 6.2 Hz)
3	130.7	5.43 (dd, 1H, *J* = 16.4, 7.0 Hz)
4	50.4	3.00 (dt, 1H, *J* = 7.0, 7.0 Hz)
5	137.6	
6	129.2	5.94 (d, 1H, *J* = 11.0 Hz)
7	126.9	6.43 (ddq,, 1H, *J* = 14.8, 11.0, 1.2 Hz)
8	129.6	5.70 (dq, 1H, *J* = 14.8, 6.7 Hz)
9	17.0	1.77 (dd, 3H, *J* = 6.7, 1.2 Hz)
10a	58.1	4.18 (d, 1H, *J* = 12.0 Hz)
10b		4.14 (d, 1H, *J* = 12.0 Hz)
11a	64.4	3.63 (dd, 1H, *J* = 10.7, 7.0 Hz)
11b		3.55 (dd, 1H, *J* = 10.7, 7.0 Hz)

### Microglial culture

The murine BV-2 microglial cell line cultured followed the procedure of our previous reports (Hsiao et al. 2020). In summary, BV-2 cells were cultured with DMEM containing Fetal Bovine Serum (FBS), streptomycin/penicillin, Lglutamine and HEPES at 37 °C, humidified 5% CO_2_. Prior to the study, BV-2 cells were cultured in FBS media (5%), pretreated with carrier media or various concentrations of compounds for 15 min, and eventually collected after 24 h of stimulation with LPS (150 ng/mL).

### Cell viability assays

As in our previous report, cellular viability was assessed using MTT test where BV-2 cells, along with other compounds were treated for 24 h (Hsiao et al. 2020).

### Detection of nitric oxide production

The level of nitric oxide (NO) metabolites from the production of activated BV-2 cells was measured with reference to the Griess method (Wang et al. 2018).
